# Combined prognostic value of lipoprotein(a) and an integrated inflammatory-lipid index in patients with acute coronary syndrome and type 2 diabetes mellitus

**DOI:** 10.3389/fcvm.2026.1811591

**Published:** 2026-06-02

**Authors:** Tingting Li, Hongliang Cong, Lin Wang, Wei Ruo, Wenyu Li

**Affiliations:** Department of Cardiology, Tianjin Chest Hospital, Tianjin, China

**Keywords:** acute coronary syndrome, inflammation, lipoprotein(a), MACE, prognosis, type 2 diabetes mellitus

## Abstract

**Background and aims:**

Lipoprotein(a) [Lp(a)] and inflammation are critical drivers of residual risk. We aimed to evaluate the combined prognostic value of Lp(a) and a novel Inflammatory-Lipid Index [defined as hs−CRP × (ApoB/ApoA1)] in patients with acute coronary syndrome (ACS) and type 2 diabetes mellitus (T2DM).

**Methods and results:**

A total of 1,910 patients with ACS and T2DM were analyzed over a median follow-up of 5.5 years. Multivariable Cox regression confirmed that both Lp(a) and the Inflammatory-Lipid Index were independent predictors of major adverse cardiovascular events (MACE). Restricted cubic splines revealed significant non-linear, positive correlations between these markers and MACE risk (both *P* for non−linearity <0.001). Patients were stratified into four subgroups based on median values. Those in the dual-high group [high Lp(a) and high Inflammatory-Lipid Index] had the highest risk, with a MACE incidence of 57.7% and an 8-fold higher risk compared to the dual-low reference (HR: 8.01; 95% CI: 4.41–14.56; *P* < 0.001). Formal interaction analysis showed that the multiplicative interaction term was not statistically significant, whereas additive interaction analysis suggested an excess joint risk on the additive scale. Addition of both biomarkers to the conventional risk model improved predictive performance and reclassification (C-statistic: 0.741; NRI: 0.569; IDI: 0.053; all *P* < 0.001). In a sensitivity analysis using 3-point MACE, the overall pattern of association remained similar.

**Conclusions:**

Concomitant elevation of Lp(a) and the Inflammatory-Lipid Index identifies a particularly high-risk phenotype among ACS patients with T2DM. This dual-biomarker approach significantly enhances cardiovascular risk stratification beyond traditional factors.

## 1 Introduction

Acute coronary syndrome (ACS) remains a dominant contributor to global cardiovascular mortality, with the prognosis being particularly dismal for patients with concomitant type 2 diabetes mellitus (T2DM). This specific subpopulation faces a significantly higher risk of recurrent major adverse cardiovascular events (MACE) (1). Although the widespread adoption of statins and PCSK9 inhibitors has facilitated the achievement of unprecedented low-density lipoprotein cholesterol (LDL-C) targets, a substantial “residual cardiovascular risk” remains (2). This phenomenon suggests that conventional lipid profiles alone are insufficient to capture the complete spectrum of atherogenic risk in diabetic ACS patients (3). The persistence of residual cardiovascular risk demonstrates that an exclusive focus on low-density lipoprotein cholesterol does not fully encompass the spectrum of risk. In type 2 diabetes, hyperglycaemia-driven oxidative stress and metabolic dysregulation foster a pro-inflammatory milieu that interacts with lipid metabolism in ways that standard laboratory panels fail to capture (4). Identifying non-traditional biomarkers that reflect these convergent pathways is critical for improving risk stratification and developing targeted secondary prevention strategies for diabetic acute coronary syndrome patients who remain at high risk despite guideline-directed medical therapy.

Lipoprotein(a) [Lp(a)] has emerged as a pivotal player in this residual risk spectrum, characterized by its pro-atherogenic, pro-inflammatory, and pro-thrombotic properties (5). Unlike LDL-C, Lp(a) levels are largely genetically determined and show limited response to traditional lifestyle or statin interventions (6). However, the predictive accuracy of Lp(a) in ACS patients with T2DM is often confounded by the patient's systemic inflammatory status and the overall balance of atherogenic lipoproteins. This balance is frequently represented by the apolipoprotein B to apolipoprotein A1 (ApoB/ApoA1) ratio, which has been shown to more accurately reflect the total burden of atherogenic particles relative to anti-atherogenic ones than traditional cholesterol measurements (7). While high-sensitivity C-reactive protein (hs-CRP) is a well-established marker of systemic inflammation, its prognostic value is significantly enhanced when evaluated in conjunction with the lipid profile (8). Despite their established roles, Lp(a), hs-CRP, and the ApoB/ApoA1 ratio are typically assessed as independent variables, without adequately capturing their combined clinical relevance. Atherogenesis is a localized inflammatory response to the entrapment of atherogenic lipoproteins within the arterial wall; therefore, assessing inflammation and lipid status in isolation may lead to an underestimation of cardiovascular risk (9). There is currently no integrated index that simplifies this complex interplay for clinical use. Specifically, whether the combined assessment of Lp(a) and an integrated inflammatory-lipid metric can improve risk stratification remains unclear. Whether such an integration could identify high-risk phenotypes among ACS patients with T2DM is a critical question that this study seeks to answer.

The present study was designed to bridge this gap by introducing a novel Inflammatory-Lipid Index—calculated as hs−CRP × (ApoB/ApoA1)—and evaluating its combined prognostic value with Lp(a) in a large retrospective cohort of 1,910 diabetic ACS patients. Over a follow-up of 5.5 years, we systematically assessed the independent and combined predictive capacities of these markers using net reclassification improvement (NRI) and integrated discrimination improvement (IDI). Our findings demonstrate that integrating the Inflammatory-Lipid Index with Lp(a) provides incremental value for MACE risk stratification.This study provides a robust clinical tool for enhanced risk stratification, potentially shifting the management of diabetic ACS toward more personalized, multi-pathway intervention strategies.

## 2 Methods

### 2.1 Ethical considerations

The study protocol was approved by the Ethics Committee of Tianjin Chest Hospital (Approval No. 2026LW-012) and conducted in adherence to the principles of the Declaration of Helsinki. Given the retrospective nature of the study, the requirement for written informed consent was waived by the Ethics Committee.

### 2.2 Study population and design

**Figure 1:**
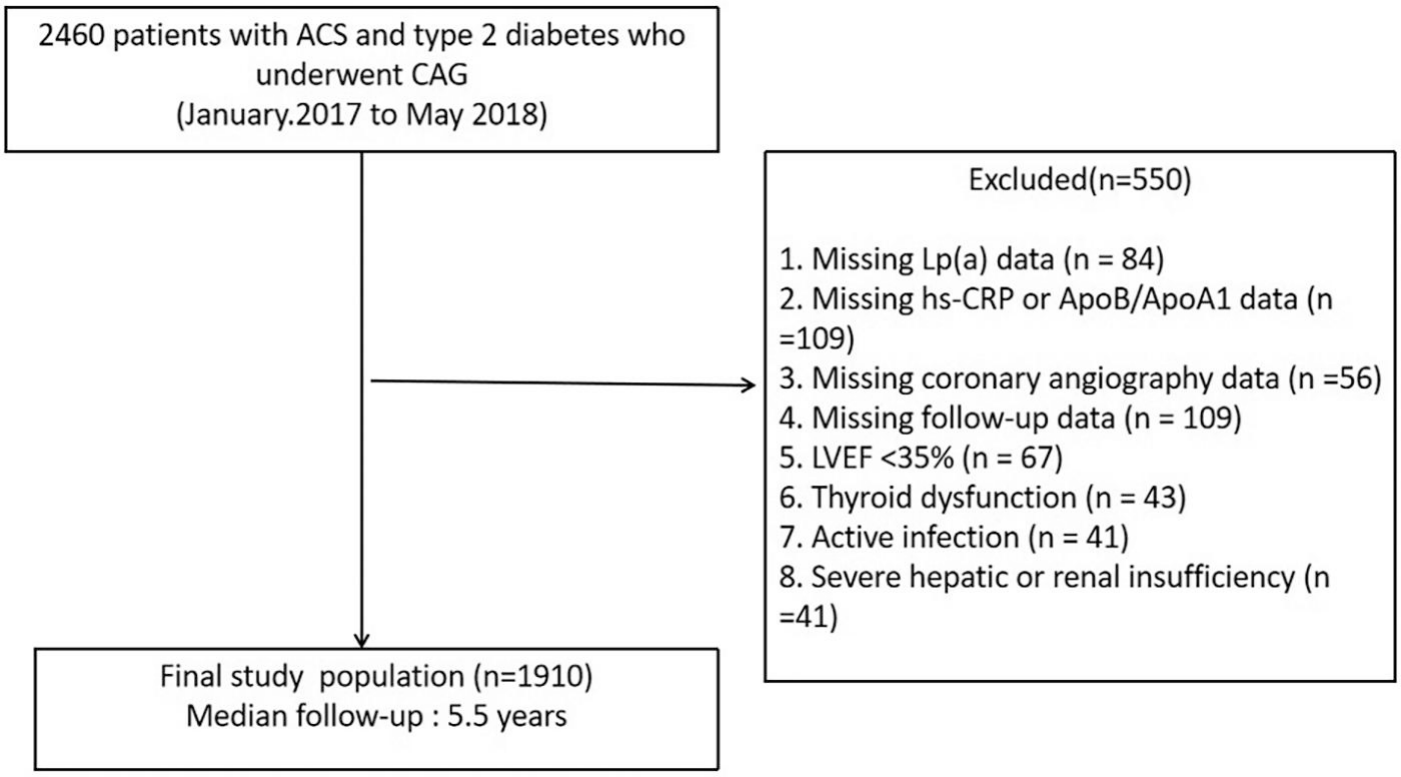
Patient flowchart.

This retrospective cohort study screened 2,460 patients with a primary diagnosis of acute coronary syndrome (ACS) admitted to the Department of Cardiology at Tianjin Chest Hospital between January 2017 and May 2018. Eligible participants were aged 18–80 years, had a confirmed diagnosis of type 2 diabetes mellitus (T2DM), presented within 72 h of symptom onset, and underwent coronary angiography. Patients were excluded based on the following criteria: (1) missing laboratory data for Lp(a), hs-CRP, or ApoB/ApoA1 (*n* = 193); (2) incomplete coronary angiography or follow-up records (*n* = 165); (3) severe hepatic or renal insufficiency (*n* = 41); (4) thyroid dysfunction (*n* = 43);(5) active infectionor and systemic inflammatory disorders (*n* = 41); (6) a left ventricular ejection fraction (LVEF) < 35% (*n* = 67). Consequently, 1,910 patients were enrolled in the final analysis (Figure 1).

### 2.3 Data collection and laboratory measurements

Clinical and demographic data were retrieved from the hospital's electronic medical record (EMR) system by trained investigators. Recorded variables included age, sex, body mass index (BMI), comorbidities (hypertension, previous cerebral infarction), lifestyle factors (smoking and alcohol consumption), and history of revascularization. Venous blood samples were obtained following an overnight fast of at least 8 h. Serum TC, TG, HDL-C, LDL-C, and FPG were measured on a Roche cobas c701 automated clinical chemistry analyzer using enzymatic assays. ApoA1 and ApoB were determined by immunoturbidimetric methods with Beckman Coulter reagents, whereas Lp(a) was assayed using the Tina-quant® immunoturbidimetric method (Roche Diagnostics, Mannheim, Germany). hs-CRP was measured by a latex-enhanced immunoturbidimetric assay on the Roche cobas platform.

### 2.4 Definitions and stratification

The primary endpoint was major adverse cardiovascular events (MACE), defined as a composite of all-cause mortality, non-fatal myocardial infarction, non-fatal stroke, and revascularization. To evaluate the combined impact of inflammation and lipid metabolism, we calculated the Inflammatory-Lipid Index as: hs-CRP × (ApoB/ApoA1). Patients were first dichotomized based on the median concentrations of Lp(a) (cut-off: 28.95 nmol/L) and the Inflammatory-Lipid Index (cut-off: 1.66). Subsequently, the cohort was stratified into four subgroups: (1) Lp(a)-L + Index-L (*n* = 518): Low Lp(a) and low Index; (2) Lp(a)-H + Index-L (*n* = 435): High Lp(a) and low Index; (3) Lp(a)-L + Index-H (*n* = 437): Low Lp(a) and high Index; (4) Lp(a)-H + Index-H (*n* = 520): High Lp(a) and high Index.

### 2.5 Statistical analysis

Continuous variables were assessed for normality using the Kolmogorov–Smirnov test. Normally distributed data are presented as mean ± standard deviation (SD) and compared using the Student's *t*-test. Non-normally distributed variables, including Lp(a), are expressed as median with interquartile range (IQR) and analyzed via the Mann–Whitney *U*-test. Categorical variables are reported as frequencies (percentages) and compared using the chi-square test or Fisher's exact test, as appropriate. Because the present study was designed to evaluate the complementary prognostic value of a genetically influenced lipoprotein marker [Lp(a)] and a prespecified integrated inflammatory-lipid marker [hs-CRP × (ApoB/ApoA1)], alternative composite indices such as hs-CRP × (LDL-C/HDL-C), hs-CRP × non-HDL-C, or higher-order products including Lp(a) were not entered into the primary model. Their potential value is acknowledged and discussed as a direction for future validation studies.

Survival outcomes were visualized using Kaplan–Meier curves, with differences between groups evaluated by the log-rank test. To identify independent predictors of MACE, multivariate Cox proportional hazards regression analysis was performed using a stepwise selection method (entry/stay criteria of 0.1/0.1). The model adjusted for potential confounders, including smoking, alcohol consumption, history of PCI/CABG, LDL-C, HDL-C, FPG, Lp(a), and the Inflammatory-Lipid Index.

Optimal cut-off values for Lp(a) and the Inflammatory-Lipid Index were determined through receiver operating characteristic (ROC) curve analysis. Restricted cubic spline analyses were performed to explore the non-linear association between the Inflammatory-Lipid Index and MACE. Four knots were placed at the 5th, 35th, 65th, and 95th percentiles of the Inflammatory-Lipid Index distribution, and the median value was used as the reference. To reduce instability caused by sparse observations at the extreme upper tail, the spline plot for the Inflammatory-Lipid Index was truncated at the 99th percentile in the main figure. Furthermore, the incremental predictive value of adding Lp(a) and the Inflammatory-Lipid Index to a baseline model (comprising smoking, alcohol consumption, HDL-C, LDL-C, and prior PCI/CABG) was evaluated using the C-statistic, net reclassification improvement (NRI), and integrated discrimination improvement (IDI).In response to concerns regarding inclusion of revascularization in the composite endpoint, an additional sensitivity analysis was performed using a 3-point MACE definition, including all-cause death, non-fatal myocardial infarction, and non-fatal stroke.

All statistical tests were two-sided, with significance defined as *P* < 0.05. Analyses were conducted using SPSS (version 25.0; IBM Corp., Armonk, NY, USA), SAS (version 9.4; Cary, NC, USA), and R (version 4.4.1; R Foundation for Statistical Computing, Vienna, Austria).

## 3 Results

### 3.1 Baseline characteristics of patients

A total of 1,910 patients with ACS and T2DM were included, with a follow-up of 5.5 years. During this period, 537 (28.1%) patients experienced MACE. The study population had a mean age of 63.2 years, 59.2% were male, and 75.4% had hypertension; these baseline demographics were balanced between the MACE and non-MACE groups (all *P* > 0.05).

Compared with the non-MACE group, patients who experienced MACE had a significantly higher prevalence of smoking (45.07% vs. 30.59%, *P* < 0.001) and a higher frequency of prior revascularization (PCI or CABG: 63.69% vs. 37.14%, *P* < 0.001). Regarding laboratory findings, the MACE group exhibited significantly elevated levels of Lp(a) [median 51.5 (IQR 27.3, 115.0) vs. 22.5 (10.7, 54.4) nmol/L, *P* < 0.001] and a higher Inflammatory-Lipid Index [median 3.3 (1.6, 7.7) vs. 1.1 (0.5, 3.3), *P* < 0.001].

Differences in metabolic indices were also observed. Patients with MACE had slightly higher FPG (8.26 ± 3.18 vs. 7.95 ± 2.85 mmol/L, *P* = 0.049), higher TC (4.65 ± 1.19 vs. 4.41 ± 1.17 mmol/L), and higher LDL-C (3.11 ± 1.06 vs. 2.85 ± 1.00 mmol/L) compared to those without MACE. Conversely, HDL-C levels were significantly lower in the MACE group (1.00 ± 0.24vs.1.04 ± 0.26 mmol/L, *P* = 0.007). No significant differences were observed between the two groups regarding BMI, alcohol consumption, history of cerebral infarction, HbA1c, or the use of secondary prevention medications, including antiplatelet agents, RAAS inhibitors, and statins (all *P* > 0.05, Table 1).

**Table 1:** Baseline characteristics of the study population.

Clinical characteristics	Overallpopulation (*n* = 1,910)	Non-MACE (*n* = 1,373)	MACE (*n* = 537)	*P*-value
Age, years	63.16 ± 8.55	63.05 ± 8.62	63.44 ± 8.38	0.367
Male, *n* (%)	1,131 (59.21%)	817 (59.50%)	314 (58.47%)	0.718
Hypertension, *n* (%)	1,440 (75.39%)	1,040 (75.75%)	400 (74.49%)	0.607
Smoking, *n* (%)	662 (34.66%)	420 (30.59%)	242 (45.07%)	<0.001
Alcohol consumption, *n* (%)	102 (5.34%)	82 (5.97%)	20 (3.72%)	0.064
BMI, kg/m^2^	25.68 ± 2.60	25.61 ± 2.62	25.78 ± 2.56	0.195
Previous cerebral infarction, *n* (%)	355 (18.59%)	245 (17.84%)	110 (20.48%)	0.205
Previous PCI/CABG, *n* (%)	852 (44.61%)	510 (37.14%)	342 (63.69%)	<0.001
Lp(a), nmol/L	28.9 (12.6, 70.8)	22.5 (10.7,54.4)	51.5 (27.3, 115.0)	<0.001
Inflammatory-Lipid Index	1.7 (0.6, 4.6)	1.1 (0.5, 3.3)	3.3 (1.6, 7.7)	<0.001
FPG, mmol/L	8.04 ± 2.95	7.95 ± 2.85	8.26 ± 3.18	0.049
TC, mmol/L	4.48 ± 1.18	4.41 ± 1.17	4.65 ± 1.19	<0.001
TG, mmol/L	1.91 ± 1.22	1.91 ± 1.29	1.89 ± 1.03	0.717
HDL-C, mmol/L	1.02 ± 0.25	1.03 ± 0.26	1.00 ± 0.24	0.007
LDL-C, mmol/L	2.92 ± 1.02	2.85 ± 1.00	3.11 ± 1.06	<0.001
VLDL-C, mmol/L	0.53 ± 0.40	0.52 ± 0.42	0.54 ± 0.34	0.425
HbA1c, %	7.89 ± 1.47	7.89 ± 1.47	7.87 ± 1.47	0.788
Medications at discharge				
Anti-platelet therapy, *n* (%)	1,832 (95.92%)	1,314 (95.70%)	518 (96.46%)	0.532
RAAS inhibitorn, *n* (%)	1,163 (60.89%)	842 (61.33%)	321 (59.78%)	0.568
Statin, *n* (%)	1,761 (92.20%)	1,257 (91.55%)	504 (93.85%)	0.111

Data are expressed as mean ± SD, medians with interquartile ranges or percentage. BMI, body mass index; PCI, percutaneous coronary intervention; CABG, coronary artery by graft; TC, total cholesterol; TG, triglycerides; HDL-C, high-density lipoprotein cholesterol; LDL-C, low-density lipoprotein cholesteass; Lp(a), lipoprotein (a); FPG, fasting plasm glucose; VLDL-C, very low-density lipoprotein cholesterol; HbA1c, Hemoglobin A1c; RAAS, renin- angiotensin -aldosterone system; MACE, major adverse cardiovascular event.

### 3.2 Associations of Lp(a) and MACE in ACS with T2DM

Univariate Cox regression analysis identified smoking, prior PCI/CABG, elevated LDL-C, low HDL-C, higher FPG, and increased levels of Lp(a) and the Inflammatory-Lipid Index as significant risk factors for MACE (all *P* < 0.05). Alcohol consumption showed a borderline protective association (HR = 0.61, *P* = 0.052), while age, sex, hypertension, history of stroke, TG, VLDL-C, and HbA1c did not exhibit significant associations.

In the multivariable model, after adjusting for potential confounders, smoking (HR = 2.02, 95% CI: 1.61–2.54; *P* < 0.001) and prior PCI/CABG (HR = 2.84, 95% *CI*: 2.28–3.53; *P* < 0.001) remained the strongest independent predictors of MACE. Each 1 mmol/L increment in LDL-C was associated with a 28% increased risk of MACE (HR = 1.28, 95% *CI*: 1.15–1.42; *P* < 0.001), whereas higher HDL-C levels conferred significant protection (HR = 0.52, 95% *CI*: 0.33–0.83; *P* = 0.006). Both Lp(a) and the Inflammatory-Lipid Index, treated as continuous variables, remained independently associated with MACE, with each unit increase corresponding to a 1% higher risk (HR = 1.01for both; *P* < 0.001 and *P* = 0.035, respectively, Table 2).

**Table 2:** Univariate and multivariate Cox regression analysis for MACE.

Variable	Univariate		Multivariate	
	HR (95% CI)	*P* value	HR (95% CI)	*P* value
Age	1.01 (0.99–1.02)	0.395		
Sex	1.04 (0.85–1.28)	0.680		
Hypertension	0.93 (0.74–1.18)	0.566		
Smoking	1.86 (1.52–2.29)	<0.001	2.02 (1.61–2.54)	<0.001
Alcohol consumption	0.61 (0.37–1.00)	0.052	0.45 (0.26–0.77)	0.003
Previous cerebral infarction	1.19 (0.92–1.52)	0.183		
Previous PCI/CABG	2.97 (2.41–3.65)	<0.001	2.84 (2.28–3.53)	<0.001
LDL-C	1.27 (1.16–1.40)	<0.001	1.28 (1.15–1.42)	<0.001
HDL-C	0.58 (0.39–0.88)	0.010	0.52 (0.33–0.83)	0.006
TG	0.99 (0.91–1.07)	0.729		
VLDL-C	1.10 (0.86–1.40)	0.465		
Lp(a) (nmol/L)	1.01 (1.01–1.01)	<0.001	1.01 (1.01–1.01)	<0.001
Inflammatory-Lipid Index	1.01 (1.01–1.02)	<0.001	1.01 (1.00–1.01)	0.035
FPG	1.04 (1.00–1.07)	0.039	1.01 (0.98–1.05)	0.422
HbA1c	0.99 (0.93–1.06)	0.788		

PCI, percutaneous coronary intervention; CABG, coronary artery bypass graft; LDL-C, low-density lipoprotein cholesterol; HDL-C, high-density lipoprotein cholesterol; TG, triglycerides; VLDL-C, very low-density lipoprotein cholesterol; Lp(a), lipoprotein (a); FPG, fasting plasm glucose; HbA1c, hemoglobin A1c; MACE, major adverse cardiovascular event; HR, hazard ratio; CI, confdential intervals.

**Figure 2:**
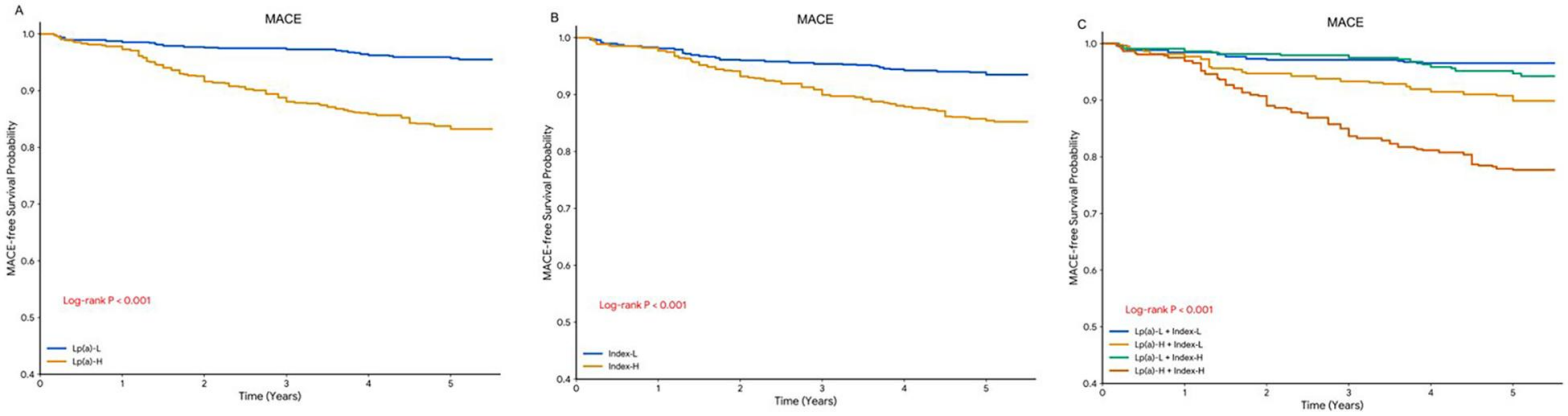
Kaplan–meier survival curves for MACE-free survival according to different biomarker levels. **(A)** MACE-free survival probability stratified by the median level of Lp(a). **(B)** MACE-free survival probability stratified by the median level of Inflammatory-Lipid Index. **(C)** MACE-free survival probability based on the combined categories of Lp(a) and the Inflammatory-Lipid Index composite factor. *P*-values were calculated using the log-rank test. MACE, major adverse cardiovascular events,Lp(a), lipoprotein (a).

Participants were categorized into two groups based on the median Lp(a) level: the Lp(a)-L group (<28.95 nmol/L, *n* = 955) and the Lp(a)-H group (≥28.95 nmol/L, *n* = 955). As summarized in Table 3, the incidence of MACE was markedly higher in the Lp(a)-H group compared to the Lp(a)-L group (40.7% vs. 15.5%, *P* < 0.001). Kaplan–Meier survival analysis confirmed that patients with higher Lp(a) levels experienced a significantly lower event-free survival rate (Log-rank *P* < 0.001; Figure 2A). After adjusting for potential confounders—including smoking, alcohol consumption, prior PCI/CABG, LDL-C, HDL-C, and FPG—multivariable Cox regression analysis identified the Lp(a)-H group as having a substantially increased risk of MACE compared to the Lp(a)-L group (HR: 3.92;95% *CI*:2.64–5.81; *P* < 0.001).

**Table 3:** Performance parameters from the ROC analysis of Lp(a), inflammatory-lipid index and their combination for predicting MACE.

Variable	Events, *n*/Total	Unadjusted HR (95% CI)	*P* value	Adjusted HR (95% CI)	*P* value
Lp(a)					
Low	148/955	1.00 (1.00–1.00)	Reference	1.00 (1.00–1.00)	Reference
High	389/955	2.93 (2.43–3.54)	<0.001	3.92 (2.64–5.81)	<0.001
Inflammatory-Lipid Index					
Low	139/953	1.00 (1.00–1.00)	Reference	1.00 (1.00–1.00)	Reference
High	398/957	3.06 (2.52–3.71)	<0.001	2.45 (1.72–3.50)	<0.001
Combined categories					
G1(Lp(a)-L + Index-L)	50/518	1.00 (1.00–1.00)	Reference	1.00 (1.00–1.00)	Reference
G2(Lp(a)-H + Index-L)	89/435	2.24 (1.59–3.17)	<0.001	3.15 (1.67–5.94)	<0.001
G3(Lp(a)-L + Index-H)	98/437	2.36 (1.68–3.31)	<0.001	1.87 (0.92–3.80)	0.065
G4(Lp(a)-H + Index-H)	300/520	7.09 (5.25–9.56)	<0.001	8.01 (4.41–14.56)	<0.001

Lp(a), lipoprotein (a); MACE, major adverse cardiovascular event; HR, hazard ratio; CI, confdential intervals; ROC, receiver operating characteristic.

**Figure 3:**
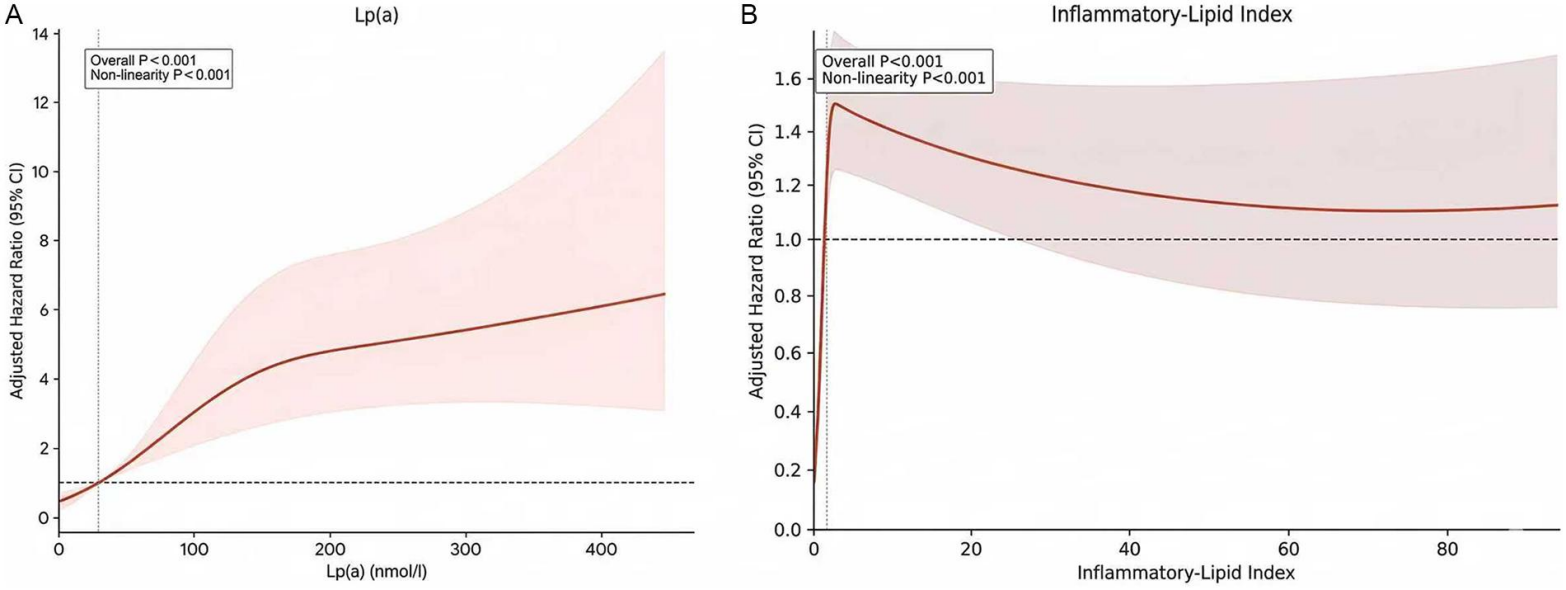
Restricted cubic spline curves for the association of Lp(a) and the inflammatory-lipid index with the risk of MACE. **(A)** Association between Lp(a) and adjusted hazard ratio for MACE. **(B)** Association between the inflammatory-lipid index and adjusted hazard ratio for MACE. The solid red line represents the adjusted hazard ratio, and the shaded area indicates the 95% confidence interval. Four knots were placed at the 5th, 35th, 65th, and 95th percentiles. The horizontal dashed line indicates HR = 1. For the Inflammatory-Lipid Index, the curve in the main figure was truncated at the 99th percentile because of sparse observations in the extreme upper tail, which may lead to instability of the spline estimates.

**Figure 4:**
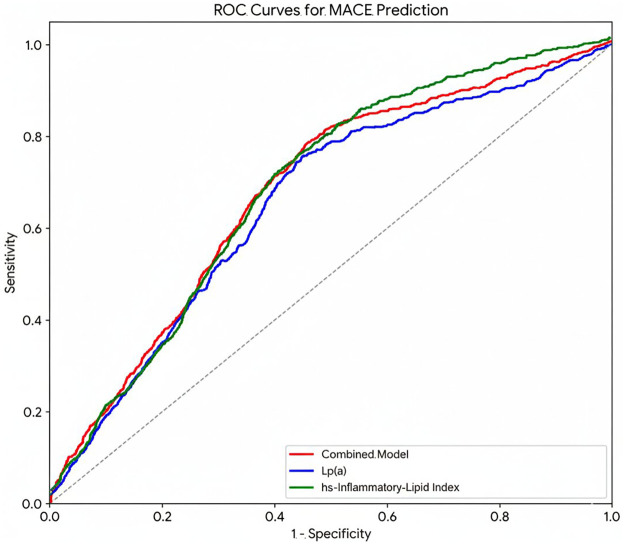
Comparison of ROC curve for Lp(a), inflammatory-lipid index and their combined model in predicting MACE. Lp(a), lipoprotein (a); MACE major adverse cardiovascular event.

To further explore the relationship between Lp(a) as a continuous variable and MACE, restricted cubic spline (RCS) curves were employed. The analysis revealed a significant positive and non-linear correlation (*P* for non−linearity <0.001; Figure 3A), suggesting a potential threshold effect. Additionally, ROC curve analysis determined an optimal cut-off value of 26.6 nmol/L for Lp(a) in predicting MACE, with an area under the curve (AUC) of 0.675 (95% CI: 0.646–0.704; *P* < 0.001). At this threshold, the sensitivity and specificity were 75.98% and 56.37%, respectively (Table 4, Figure 4).

**Table 4:** ROC curve for Lp(a), inflammatory-lipid Index and their combination in predicting MACE.

Variable	AUC	Optimal cut-off value	Sensitivity %	Specificity%	95% CI	*P* value
Lp(a)	0.675	26.60	75.98	56.37	0.646–0.704	<0.001
Inflammatory-Lipid Index	0.702	1.62	75.42	58.49	0.677–0.726	<0.001
Lp(a) + Inflammatory-Lipid Index	0.698		79.14	55.86	0.670–0.723	<0.001

Lp(a), lipoprotein (a); MACE, major adverse cardiovascular event; ROC, receiver operating characteristic; AUC, an area under the cure; CI, confidential intervals.

### 3.3 Predictive value of the inflammatory-lipid index for MACE in ACS with T2DM

Similar to the findings for Lp(a), the cohort was stratified based on the median Inflammatory-Lipid Index into the Index-L (<1.66, *n* = 953) and Index-H (≥1.66, *n* = 957) groups. Patients in the Index-H group experienced a substantially higher incidence of MACE compared to those in the Index-L group (41.6% vs. 14.6%, *P* < 0.001; Table 3). This divergence in clinical outcomes was further supported by Kaplan–Meier analysis, which demonstrated that an elevated Inflammatory-Lipid Index was significantly associated with diminished event-free survival (Log-rank *P* < 0.001; Figure 2B). After multivariable adjustment for potential confounders, the Index-H group maintained a robust and independent association with MACE risk, yielding an adjusted HR of 2.45 (95% *CI*: 1.72–3.50, *P* < 0.001).

Restricted cubic spline analysis showed a significant non-linear association between the Inflammatory-Lipid Index and MACE (*P* for non−linearity <0.001; Figure 3B). However, the apparent decline in hazard estimates at the extreme upper tail should be interpreted with caution, as this likely reflects sparse data and instability of the spline fit in that range rather than a true protective association. To improve interpretability, the spline plot shown in the main figure was truncated at the 99th percentile. Only 20 patients (1.05%) had Inflammatory-Lipid Index values above the 99th percentile. Furthermore, ROC curve analysis identified an optimal threshold of 1.62 for the Index in predicting MACE, with an AUC of 0.702 (95% CI:0.677–0.726, *P* < 0.001). At this cut-off value, the sensitivity and specificity were 75.42% and 58.49%, respectively (Table 4, Figure 4). Of note, in the initial ROC analysis (Table 4), the AUC for the Inflammatory-Lipid Index (0.702) was numerically similar to that of the raw combined model (0.698), with overlapping 95% confidence intervals indicating no statistically significant difference in their independent discriminative power at this stage. To more rigorously evaluate the synergistic value of these markers beyond such univariate combinations, we further integrated them into a comprehensive multivariable clinical model (see Section 3.7 and Table 6).

### 3.4 Combined impact of Lp(a) and the inflammatory-lipid index on MACE

To evaluate the combined association between Lp(a) and the Inflammatory-Lipid Index, patients were stratified into four mutually exclusive subgroups based on their respective median values: G1 [Lp(a)-L + Index-L, *n* = 518], G2 [Lp(a)-H + Index-L, *n* = 435], G3 [Lp(a)-L + Index-H, *n* = 437], and G4 [Lp(a)-H + Index-H, *n* = 520].

The incidence of MACE across these groups was 9.7%, 20.5%, 22.4%, and 57.7%, respectively (*P* < 0.001; Table 3). Compared with the reference group (G1), groups G2, G3, and G4 showed a stepwise increase in MACE risk. After adjusting for potential confounders, the risk of MACE remained significantly elevated in G2 (HR: 3.15; 95% *CI*: 1.67–5.94, *P* < 0.001) and G4 (HR: 8.01; 95% *CI*: 4.41–14.56, *P* < 0.001). Group G3 exhibited a 1.87-fold increased risk, though this association was borderline significant in the fully adjusted model (95% *CI*: 0.92–3.80, *P* = 0.065).

**Figure 5:**
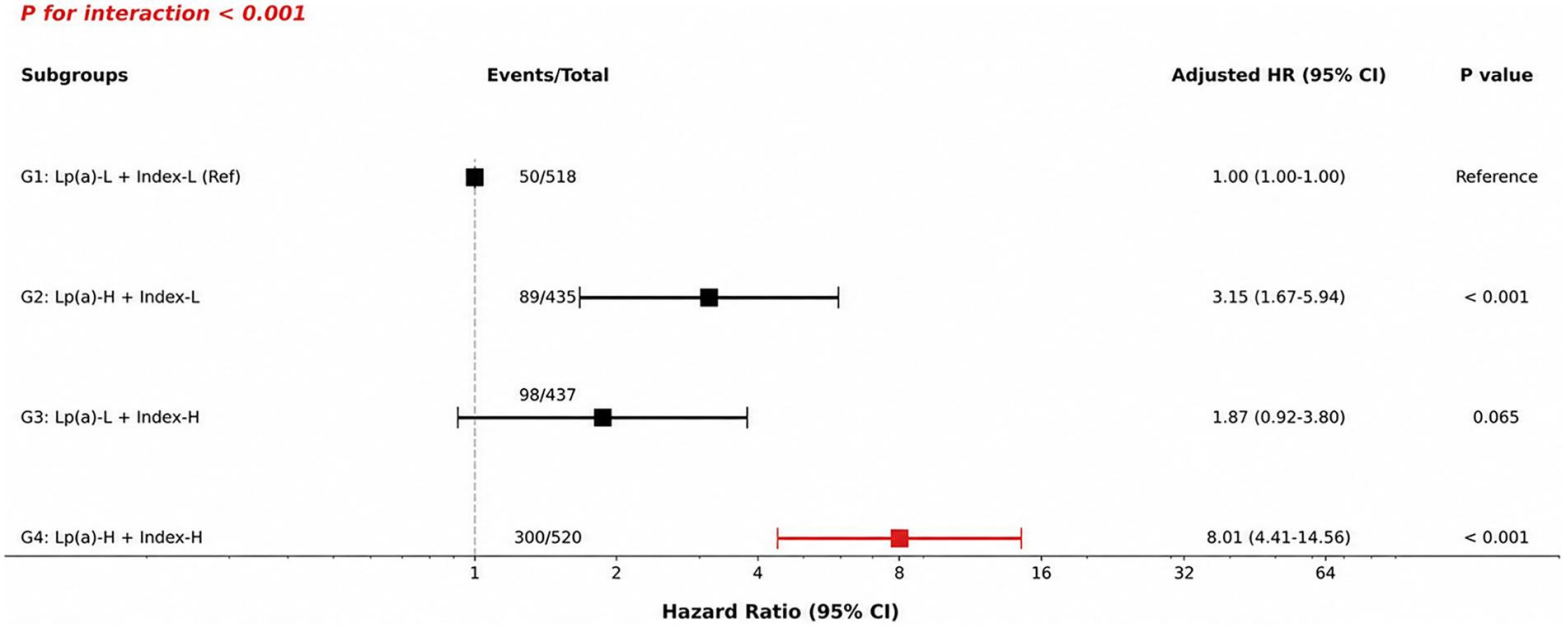
Combined impact of lipoprotein(a) and the inflammatory-lipid index on long-term MACE risk. Subgroup analysis and forest plot of adjusted hazard ratios for major adverse cardiovascular events (MACE). Patients were stratified into four phenotypic categories based on the median values of Lp(a) and the novel inflammatory-lipid index. Group 1 (G1, Reference): low Lp(a) and low inflammatory-lipid index. Group 2 (G2): High Lp(a) and low inflammatory-lipid index. Group 3 (G3): Low Lp(a) and high inflammatory-lipid index. Group 4 (G4): high Lp(a) and high inflammatory-lipid index. The black and red squares represent the point estimates of the Hazard Ratios (HRs), and the horizontal lines represent the 95% confidence intervals (CIs).

As illustrated by the Kaplan–Meier survival curves, the G4 group [concomitant high Lp(a) and high Inflammatory-Lipid Index] exhibited the lowest event-free survival rate among the four groups (Log-rank *P* < 0.001), indicating that patients with concomitant elevation of both biomarkers had the highest long-term risk of MACE (Figure 2C and Figure 5).

### 3.5 Formal interaction analysis between Lp(a) and the inflammatory-lipid index

To further examine the combined effect of Lp(a) and the Inflammatory-Lipid Index on MACE risk, formal interaction analyses were performed on both the multiplicative and additive scales. In the multivariable Cox proportional hazards model adjusted for smoking, alcohol consumption, prior PCI/CABG, LDL-C, HDL-C, and FPG, elevated Lp(a) and elevated Inflammatory-Lipid Index were both independently associated with an increased risk of MACE. The adjusted hazard ratio was 2.23 (95% CI: 1.58–3.16) for elevated Lp(a) and 2.13 (95% CI: 1.51–3.00) for elevated Inflammatory-Lipid Index. The multiplicative interaction term between the two variables was not statistically significant (HR: 1.27, 95% CI: 0.84–1.93; *P* = 0.257).

Additive interaction was then assessed using the relative excess risk due to interaction (RERI), attributable proportion (AP), and synergy index (SI). The RERI was 2.68 (95% CI: 1.61–3.76), the AP was 0.44 (95% CI: 0.31–0.58), and the SI was 2.14 (95% CI: 1.39–2.89). These findings suggest a positive additive interaction between elevated Lp(a) and elevated Inflammatory-Lipid Index.

### 3.6 Sensitivity analysis using 3-point MACE

A sensitivity analysis was performed using 3-point MACE, defined as all-cause death, non-fatal myocardial infarction, and non-fatal stroke. Using the same cut-off values for Lp(a) and the Inflammatory-Lipid Index as in the primary analysis, 288 events were identified during follow-up. After adjustment for smoking, alcohol consumption, prior PCI/CABG, LDL-C, HDL-C, and FPG, elevated Lp(a) and elevated Inflammatory-Lipid Index were both associated with a higher risk of 3-point MACE. The adjusted hazard ratio was 3.27 (95% CI: 1.99–5.37; *P* < 0.001) for elevated Lp(a) and 2.99 (95% CI: 1.81–4.92; *P* < 0.001) for elevated Inflammatory-Lipid Index. The interaction term between the two variables was not statistically significant (HR: 0.74, 95% CI: 0.41–1.32; *P* = 0.302).

Patients were then classified into four groups according to the combined status of Lp(a) and the Inflammatory-Lipid Index. Compared with the group with low levels of both markers, the adjusted hazard ratio for 3-point MACE was 3.27 (95% CI: 1.99–5.37; *P* < 0.001) in the high Lp(a)/low Index group, 2.99 (95% CI: 1.81–4.92; *P* < 0.001) in the low Lp(a)/high Index group, and 7.20 (95% CI: 4.57–11.35; *P* < 0.001) in the high Lp(a)/high Index group. Overall, the sensitivity analysis showed a pattern similar to that observed in the primary analysis.

### 3.7 Incremental predictive value of Lp(a) and the inflammatory-lipid Index

To assess the clinical utility of these markers, we evaluated their incremental predictive value when added to a baseline model comprising smoking, alcohol consumption, HDL-C, LDL-C, and prior PCI/CABG. The baseline model yielded a C-statistic of 0.695 (95% *CI*: 0.675–0.715). As shown in Table 5, incorporating Lp(a) into the baseline model significantly improved model discrimination, with the C-statistic rising to 0.738 (95% *CI*: 0.716–0.760; *P* < 0.001). This addition also resulted in significant improvements in reclassification, with a net reclassification improvement (NRI) of 0.495 and an integrated discrimination improvement (IDI) of 0.047 (both *P* < 0.001). Similarly, adding the Inflammatory-Lipid Index to the baseline model also resulted in a modest yet significant improvement in the C-statistic (0.699, *P* = 0.040) and reclassification indices (NRI 0.182, IDI 0.006, both *P* < 0.001) (Table 5).

**Table 5:** Additional predictive value provided by Lp(a) and inflammatory-lipid index for predicting MACE.

Model	C-Statistic (95% CI)	*P* value	NRI (95% CI)	*P* value	IDI (95% CI)	*P* value
Original model	0.695 (0.675–0.715)	Reference	—	—	—	—
Original model + Lp(a)	0.738 (0.716–0.760)	<0.001	0.495 (0.377–0.570)	<0.001	0.047 (0.027–0.069)	<0.001
Original model + Inflammatory-Lipid Index	0.699 (0.680–0.725)	0.040	0.182 (0.077–0.282)	<0.001	0.006 (0.001–0.018)	<0.001
Original model + Lp(a) + Inflammatory-Lipid Index	0.741 (0.720–0.763)	<0.001	0.569 (0.435–0.703)	<0.001	0.053 (0.027–0.079)	<0.001

Lp(a), lipoprotein (a); MACE, major adverse cardiovascular event; NRI, net reclassifcation improvement; IDI, integrated discrimination improvement; CI, confdential intervals. Original model included smoking, alcohol consumption, HDL-C, LDL-C, previous PCI/CABG.

Most importantly,when both Lp(a) and the Inflammatory-Lipid Index were added to the baseline model, the C-statistic increased to 0.741 (95% *CI*: 0.720–0.763; *P* < 0.001), with total NRI of 0.569 and IDI of 0.053 (all *P* < 0.001). According to Table 6, adding the Inflammatory-Lipid Index to a model already containing Lp(a) was also associated with significant improvements in the C-statistic (*P* = 0.030), NRI (0.164, *P* < 0.001), and IDI (0.006, *P* < 0.001). These findings indicate that combined assessment of Lp(a) and the Inflammatory-Lipid Index improves risk stratification for MACE beyond conventional cardiovascular risk factors. Although the standalone ROC analysis in Table 4 showed similar AUC values for the Index and the combined markers, the multivariable incremental analysis (Table 6) confirms that the dual-biomarker approach provides superior clinical utility by significantly improving the baseline model's discrimination (*P* = 0.030 for the increment).

**Table 6:** Additional predictive value after the addition of Lp(a) or inflammatory-lipid index to original model containing the other marker.

Model	C-statistic (95% CI)	*P* value	NRI (95% CI)	*P* value	IDI (95% CI)	*P* value
Original model + Inflammatory-Lipid Index	0.699 (0.680–0.725)					
Lp(a) + original model + Inflammatory-Lipid Index	0.741 (0.720–0.763)	<0.001	0.495 (0.374–0.571)	<0.001	0.046 (0.026–0.068)	<0.001
Original model + Lp(a)	0.738 (0.716–0.760)					
Inflammatory-Lipid Index + original model + Lp(a)	0.741 (0.720–0.763)	0.030	0.164 (0.068–0.272)	<0.001	0.006 (0.001–0.016)	<0.001

Lp(a), lipoprotein (a); MACE, major adverse cardiovascular event; NRI, net reclassifcation improvement; IDI, integrated discrimination improvement; CI, confdential intervals. Original model included smoking, alcohol consumption, HDL-C, LDL-C, previous PCI/CABG.

## 4 Discussion

In this large-scale retrospective cohort study of patients with acute coronary syndrome (ACS) and type 2 diabetes mellitus (T2DM), we demonstrated that elevated lipoprotein(a) [Lp(a)] and a novel Inflammatory-Lipid Index—defined as hs−CRP × (ApoB/ApoA1)—are independent predictors of long-term major adverse cardiovascular events (MACE). An important finding of the present study is that patients with concomitantly elevated Lp(a) and Inflammatory-Lipid Index had the highest long-term risk of MACE. Compared with those with low levels of both markers, this group showed an approximately 8-fold higher risk of MACE, highlighting the clinical value of combined assessment of these two biomarkers in identifying a particularly high-risk phenotype within the diabetic ACS population.

The identification of Lp(a) as an independent predictor in our cohort aligns with the growing body of evidence characterizing it as a key component of residual cardiovascular risk. While LDL-C remains the primary target of therapy, our results confirm that Lp(a) provides prognostic information that traditional lipid panels fail to capture (10). This is consistent with the findings from the Odyssey Outcomes trial, which suggested that Lp(a) predicts cardiovascular risk even when LDL-C is aggressively lowered by PCSK9 inhibitors (11). However, our study extends these observations by specifically focusing on the T2DM population, where glucose-induced metabolic shifts may further amplify the pro-thrombotic and pro-inflammatory properties of the Lp(a) particle (5). The non-linear relationship revealed by our restricted cubic spline (RCS) analysis suggests a potential threshold effect, implying that clinical risk escalates rapidly once Lp(a) exceeds a median level of approximately 26–29 nmol/L.

In this study, we introduced and validated the Inflammatory-Lipid Index, which integrates hs-CRP and the ApoB/ApoA1 ratio to capture both systemic inflammation and lipid imbalance in patients with ACS and T2DM. The choice of a multiplicative formula [hs-CRP × (ApoB/ApoA1)] was intentionally designed to capture the non-linear, combined interaction between inflammation and lipid metabolism. While the ApoB/ApoA1 ratio represents the burden of atherogenic particles, hs-CRP acts as a catalytic factor that accelerates the transition of these particles into vulnerable plaques. This mathematical integration better reflects the “lipid-driven, inflammation-amplified” nature of atherosclerosis than individual markers used in isolation. We acknowledge that hs-CRP measured during ACS admission can be influenced by the acute phase response. However, to mitigate this, we applied strict exclusion criteria for clinical infections and systemic inflammatory disorders. Furthermore, our findings show that even when measured in the periprocedural period, the Index maintains strong long-term prognostic value, suggesting it captures a meaningful “inflammatory set-point” that is relevant to the patient's long-term cardiovascular trajectory. Previous research, such as the INTERHEART study, established the ApoB/ApoA1 ratio as a superior predictor of myocardial infarction compared to conventional cholesterol ratios (7). By incorporating hs-CRP, our index captures inflammatory residual risk, as highlighted by the CANTOS and COLCOT trials, which demonstrated that reducing inflammation can lower cardiovascular event rates independently of lipid levels (12, 13). From a practical perspective, apoB, apoA1, and hs-CRP are routinely available in many tertiary hospitals and major clinical laboratories. Accordingly, the present index should be viewed as a pragmatic tool for risk stratification in selected high-risk patients with ACS and T2DM, rather than as a basis for universal screening. While the Inflammatory-Lipid Index alone showed a significant but modest improvement over the original model (C-statistic 0.699, *P* = 0.040), its primary clinical utility is realized when combined with Lp(a). This synergy is particularly evident in the dual-high group, which exhibited a vastly higher MACE risk, supporting a strategy of integrated risk stratification.

The association between the Inflammatory-Lipid Index and MACE risk may be rooted in the complex metabolic cross-talk between systemic inflammation and lipoprotein kinetics. Mechanistically, the ApoB/ApoA1 ratio serves as a precise surrogate for the numerical balance between pro-atherogenic particles and anti-atherogenic reverse cholesterol transport (14). When this ratio is elevated, an excess of ApoB-containing lipoproteins—particularly small dense LDL particles—penetrates the arterial intima. However, the progression from simple lipid deposition to a clinically unstable lesion is largely dictated by the prevailing inflammatory milieu, represented in our study by hs-CRP (15). Assessment of these pathways in combination may therefore provide a more clinically relevant estimate of residual risk than either component alone.

This interaction can be framed within the “seed and soil” hypothesis of atherosclerosis. A high ApoB/ApoA1 ratio provides the “seeds” in the form of retained atherogenic lipoproteins, while elevated hs-CRP reflects a more permissive inflammatory “soil”. Previous studies have demonstrated that CRP can bind to oxidized LDL and activate the complement pathway, thereby promoting macrophage recruitment and amplifying local vascular inflammation (16). This lipid-driven and inflammation-amplified process may accelerate necrotic core expansion and weaken the fibrous cap through increased matrix metalloproteinase activity (17). In addition, ApoA1 not only reflects reverse cholesterol transport capacity, but is also closely related to the antioxidant and anti-inflammatory functions of HDL particles, which contribute to the anti-atherogenic properties of the HDL system. In the diabetic setting, hyperglycemia-induced oxidative stress may further intensify this process by promoting glycation of ApoB-containing particles and sustaining inflammatory cytokine release, thereby impairing ApoA1 function and cholesterol efflux (18). Taken together, these mechanisms support the biological plausibility of combining inflammatory and lipid-related markers for risk assessment in diabetic ACS patients.

One of the main contributions of the present study is the demonstration that combined elevation of Lp(a) and the Inflammatory-Lipid Index identifies a subgroup with particularly high cardiovascular risk. Patients with concomitantly high values experienced a 57.7% event rate and an 8-fold higher adjusted hazard compared with the dual-low group. This pattern is clinically relevant because it suggests that the coexistence of an elevated Lp(a) burden and an adverse inflammatory-lipid profile marks a group with both high plaque burden and increased plaque vulnerability.

Formal interaction analysis further refined this interpretation. In the present study, the multiplicative interaction term between elevated Lp(a) and elevated Inflammatory-Lipid Index was not statistically significant, whereas additive interaction analysis suggested a positive interaction. This finding indicates that the joint effect of these two markers may exceed the sum of their individual effects on the additive scale, even though a significant multiplicative interaction was not demonstrated. From a clinical perspective, this distinction is important because additive interaction may be more relevant to risk stratification and identification of vulnerable subgroups than multiplicative interaction alone.

Previous studies have examined the combined prognostic relevance of Lp(a) and inflammatory markers, although relatively few have incorporated a lipid-inflammation composite index. A prospective cohort of acute myocardial infarction reported that concurrent elevations of Lp(a) and hs-CRP approximately doubled the risk of cardiovascular and all-cause mortality (9). Similarly, data from the Multi-Ethnic Study of Atherosclerosis suggested that risk was greater when Lp(a) elevation occurred in the presence of an adverse inflammatory profile (19). Our findings extend this concept to diabetic ACS patients by showing that combined assessment of Lp(a) and the Inflammatory-Lipid Index improves identification of patients at particularly high risk. In the present study, the analysis demonstrated a clear, stepwise improvement in model performance. The C-statistic increased from 0.695 in the baseline model to 0.741 when both markers were added. Furthermore, adding the Inflammatory-Lipid Index to the Lp(a)-inclusive model significantly improved the C-statistic (*P* = 0.030), NRI, and IDI, demonstrating the incremental prognostic value of combining these biomarkers for residual risk stratification. At the same time, we acknowledge that other integrated approaches may also be informative. We did not test alternative combinations such as hs-CRP × (LDL-C/HDL-C), hs-CRP × non-HDL-C, or a single higher-order composite incorporating Lp(a), because our prespecified objective was to evaluate whether Lp(a) and the inflammatory-lipid axis provide complementary rather than merged prognostic information. Future studies should directly compare these candidate indices to determine the most parsimonious and clinically useful strategy.

In addition, the sensitivity analysis using 3-point MACE yielded results broadly similar to those of the primary analysis. After excluding revascularization from the composite endpoint, elevated Lp(a) and elevated Inflammatory-Lipid Index remained significantly associated with adverse outcomes, and the dual-high group continued to show the highest risk. These findings suggest that the association observed in the primary analysis was not solely driven by repeat revascularization events. This supplementary analysis strengthens the robustness of the main findings and supports the clinical relevance of these two biomarkers in identifying high-risk patients with ACS and T2DM.

Despite these strengths, several limitations must be acknowledged. First, the retrospective nature of this study, conducted at a single center, may introduce selection bias and limits the generalizability of our findings to other ethnic or geographical populations. Second, while we adjusted for multiple confounders, including smoking, hypertension, and prior revascularization, the potential for unmeasured confounding—such as genetic predispositions or specific dietary patterns—remains. Third, Lp(a) and the Inflammatory-Lipid Index were measured at a single time point upon admission; therefore, we could not assess how temporal changes in these markers, particularly in response to subsequent glycemic control or anti-inflammatory therapies, might influence long-term prognosis. Fourth, although the component biomarkers used to construct the Inflammatory-Lipid Index are clinically obtainable, we did not perform a formal cost-effectiveness analysis and therefore cannot conclude that routine measurement of apoB and apoA1 for all patients is justified on economic grounds. Fifth, alternative inflammatory-lipid combinations and a single merged composite incorporating Lp(a) were not tested in the current dataset. In addition, although additive interaction was observed, the multiplicative interaction term was not statistically significant. Therefore, the present findings should be interpreted as supporting the combined prognostic value of these biomarkers rather than a formally confirmed multiplicative interaction. Finally, the spline curve for the Inflammatory-Lipid Index should be interpreted with caution at the extreme upper tail, where sparse data may have contributed to instability of risk estimates.

Looking forward, these results provide a rationale for considering both Lp(a) and integrated inflammatory-lipid markers in the risk assessment of diabetic ACS patients. Combined assessment of these pathways may help refine secondary prevention strategies by identifying patients with persistently high residual risk despite guideline-directed therapy.Future prospective studies are needed to determine whether interventions targeting Lp(a), inflammation, or both can improve outcomes in the high-risk subgroup identified in this study.

## 5 Conclusion

In summary, this study demonstrates that elevated lipoprotein(a) and the novel Inflammatory-Lipid Index (hs−CRP × ApoB/ApoA1) are independent predictors of long-term MACE in patients with ACS and T2DM. Patients with concomitant elevation of both biomarkers exhibite the highest risk of adverse outcomes, with approximately an 8-fold increase in risk compared to those with low levels of both markers. While no significant multiplicative interaction was observed, additive interaction analysis revealed an excess joint risk, suggesting a synergistic impact on the additive scale. Notably, the integration of these two biomarkers provided superior incremental predictive value and improved risk reclassification compared to conventional risk factors or either marker alone. These findings support the clinical utility of a dual-biomarker approach to refine risk stratification and may guide more targeted secondary prevention strategies for diabetic ACS patients with high residual cardiovascular risk.

## Data Availability

The raw data supporting the conclusions of this article will be made available by the authors, without undue reservation.
